# Antinociceptive and anti-inflammatory activities of a triterpene-rich fraction from *Himatanthus drasticus*

**DOI:** 10.1590/1414-431X20197798

**Published:** 2019-05-16

**Authors:** S.C.X. de-Almeida, Â.C.F. da-Silva, N.R.T. Sousa, I.H.F. Amorim, B.G. Leite, K.R.T. Neves, J.G.M. Costa, C.F.B. Felipe, G.S. de-Barros Viana

**Affiliations:** 1 Departamento de Química Biológica, Universidade Regional do Cariri (URCA), Crato, CE, Brasil; 2 Laboratório de Farmacologia, Faculdade de Medicina Estácio de Juazeiro do Norte, Juazeiro do Norte, CE, Brasil; 3 Departamento de Fisiologia e Farmacologia, Faculdade de Medicina da Universidade Federal do Ceará (UFC), Fortaleza, CE, Brasil; 4 Departamento de Biologia Molecular, Universidade Federal da Paraíba, João Pessoa, PB, Brasil

**Keywords:** Pentacyclic triterpenes, Lupeol, Alpha-amyrin, Beta-amyrin, Inflammation, Himatanthus drasticus

## Abstract

*Himatanthus drasticus* (Mart.) Plumel belongs to the Apocynaceae family and the latex from its trunk bark (*Hd*) is known as “janaguba milk”. This latex is widely used in Northeast Brazil, mainly in the Cariri region, for its gastroprotective, anti-inflammatory, and antitumor properties. The objective of this study was to investigate a triterpene-rich fraction (FJNB) from *H. drasticus* latex on acute models of nociception and inflammation and to clarify its mechanisms of action. Wistar rats or Swiss mice were subjected to the carrageenan-induced paw edema test or the formalin test, respectively, after the acute oral treatment with FJNB. The inflamed paws from the carrageenan-induced paw edema and formalin tests were processed for histological and immunohistochemical assays, respectively. The results were analyzed by ANOVA and considered significant at P<0.05. FJNB (10 mg/kg) decreased the paw edema by 25% at the 3rd h after the carrageenan injection. Indomethacin, used as reference, inhibited the paw edema by 59% at the same time-point. In the formalin test, FJNB inhibited the 1st phase by 27, 49, and 52% and the 2nd phase by 37, 50, and 67%, at the doses of 1, 5, and 10 mg/kg, respectively. In addition, FJNB significantly inhibited the expressions of inducible nitric oxide synthase (iNOS), cyclooxygenase-2 (COX-2), and the inflammatory cytokine tumor necrosis factor (TNF)-alpha. The histone deacetylase (HDAC) expression and the transcription factor nuclear factor kappa (NF-kB) were also inhibited at the same doses. In conclusion, the FJNB inhibitory actions on iNOS, COX-2, TNF-α, HDAC, and NF-kB could be involved with the drug anti-inflammatory activity.

## Introduction

*Himatanthus drasticus* (Mart.) Plumel (Apocynaceae), known as “Janaguba”, is a lactifer species largely distributed in several Brazilian regions, mainly in the Cariri region (Northeast Brazil) where it can be found in the “Chapada do Araripe” (Araripe Plateau), located in the Southern region of the State of Ceará.

*H. drasticus* latex, when added to water, is popularly known as “janaguba milk” and became largely used in folk medicine for the treatment of neoplasias, after medical reports of its efficacy for lung and lymphatic cancers in the 1970s. However, janaguba milk is also used nowadays for the treatment of gastric ulcers, diabetes, inflammatory disease, and as wound healing, among other illnesses (1–3).

The pharmacological potential of the species has been demonstrated in different *in vivo* and *in vitro* pre-clinical studies, pointing to the presence of anti-inflammatory, antinociceptive, antitumor, and gastroprotective actions in the latex, bark, and leaves (4,5). Lupeol and its esters have been identified in *H. drasticus* and shown to present antinociceptive and anti-inflammatory effects (6). Although the study by Lucetti et al. (6) attributed these effects to the triterpene lupeol acetate, isolated from the latex, others described these effects in the protein fraction obtained from the latex mixture with water (janaguba milk) which, however, is devoid of lupeol.

The anti-inflammatory and antinociceptive actions of the protein fraction from the latex were reflected in the effects observed in experimental arthritis models, where the dose of 50 mg/kg *iv* reduced cell influx, myeloperoxidase activity, nitric oxide levels, inflammatory cytokines (interleukin (IL)-1β and IL-6), and edema caused by zymosan-induced arthritis (7,8). The presence of triterpene β-amyrin, condensed proanthocyanidins, and leucocyanohydrins (9,10) was also observed in the preliminary phytochemical investigation of the extract.

Pharmacological studies carried out focused on *H. drasticus*, in their majority, on the species’ anticancer and anti-inflammatory actions. However, almost none of them clearly describes the mechanisms by which *H. drasticus* exerts its actions. Thus, the objective of the present study was to evaluate the antinociceptive and anti-inflammatory effects of the triterpene-rich fraction (FJNB) isolated from *H. drasticus* latex in experimental models of nociception and inflammation. Furthermore, in order to clarify the action mechanisms involved with the observed effects of FJNB, immunohistochemical assays for inflammatory mediators (inducible nitric oxide synthase (iNOS) and cyclooxygenase-2 (COX-2)) and the pro-inflammatory cytokine tumor necrosis factor (TNF)-alpha, were carried out. In addition, the expression of histone deacetylase (HDAC) and nuclear factor kappa (NF-kB) were also measured in the formalin-inflamed paw test.

## Material and Methods

### Sample collection

Janaguba latex was obtained in the Araripe Plateau, municipality of Crato, State of Ceará, Brazil, in October 2013, by a specialized supplier according to the Brazilian Institute for the Environment and Natural Resources (IBAMA). A specimen exsiccate was deposited in the Dárdamo de Andrade Lima Herbarium of the Regional University of Cariri (URCA), under the number 10,103.

### Fraction isolation procedure

A latex suspension was prepared in distilled water (1:4), followed by filtration and centrifugation at 2500 *g* for 5 min at room temperature. The supernatant was extracted (3×50 mL) with the solvents hexane, chloroform, and ethyl acetate. The fractions obtained were concentrated under reduced pressure (15 mmHg) on a rotary evaporator, resulting in three fractions: 0.53 g (in hexane), 0.13 g (in chloroform), and 0.30 g (in ethyl acetate). The solid residue from the centrifugation was treated with 400 mL n-butanol to separate the polymer mixture. The solution was then extracted with chloroform (1:1, 3×100 mL), filtered over Na_2_SO_4_ and concentrated on a rotary evaporator, yielding a precipitate, which, after suspension in acetone (3×100 mL) and distillation, produced a white mass (3.54 g) named FJNB, soluble in pyridine and with thin layer chromatography (TLC) profile showing a single spot. FJNB was dissolved in an aqueous solution of 1% DMSO immediately before use.

### Chemical procedure for FJNB spectra

NMR spectra for FJNB were recorded on a Brüker-Avance DRX-500 model spectrometer (Billerica, USA), in pyridine, at 500 MHz for ^1^H NMR and 125 MHz for ^13^C NMR spectra. TLC was performed with plastic-backed plates coated with silica gel (1.05735, 60 A°) that were visualized by spraying with a vanillin/H_2_SO_4_ solution, followed by warming and exposure to iodine vapor.

### Animals

Male Swiss mice (62 animals, 30 g) and male Wistar rats (27 animals, 200 g) were obtained from the Animal House of the Faculty of Medicine Estácio of Juazeiro do Norte (Estácio/FMJ). The animals were maintained at 24±2°C and a 12 h dark/12 h light cycle, with standard food and water *ad libitum*. The study was approved (No. 0070/2013.2) by the Ethics Committee for Animal Experimentation of the Regional University of Cariri. All experiments followed the ethical principles established in the Guide for the Care and Use of Laboratory Animals, USA, 2011.

### Drugs and reagents

Carrageenan (lambda type IV) and indomethacin were purchased from Sigma Chemical Co. (USA), while morphine was from Cristália (Brazil). Antibodies for immunohistochemistry assays were purchased from Santa Cruz Biotechnology (USA) or Merck-Millipore Corporation (USA). All other reagents were of analytical grade.

### Carrageenan-induced paw edema test in rats

The animals were treated by gavage with FJNB (5 and 10 mg/kg). The control group was administered saline (0.1 mL/10 g). Indomethacin (INDO, 10 mg/kg) was used as reference. Thirty minutes later, edema was induced by the subplantar injection of 50 μL carrageenan (1%) into the animal's right hind paw. Measurements of the paw volume were done by means of a plethysmometer (Ugo Basile, Italy) immediately prior to the carrageenan injection and after 1, 2, 3, 4, and 24 h. The paw edema volume (mL) was determined by the difference between the final and initial volumes. The data are shown at the 3rd h, corresponding to the maximum effect.

### Formalin test in mice

Twenty microliters of 1% formalin were administered by the subplantar route in the mouse's right hind paw. The licking time was recorded from 0 to 5 min (phase 1, neurogenic) and from 20 to 25 min (phase 2, inflammatory) after the formalin injection. The animals were treated with saline (Control, 0.1 mL/10 g, *ip*), morphine (5 mg/kg, *ip*), or FJNB (10, 25, and 50 mg/kg, *ip*) 30 min before the formalin injection. The licking time (s) was measured for 5 min, at the 5th min (1st phase, neurogenic), and 20 min later (2nd phase, inflammatory).

### Hematoxylin/eosin (HE) histological analyses

Edematous paws from rats subjected to the carrageenan-induced paw edema test were fixed in buffered formalin for 24 h, followed by 75% alcohol solution. After fixation, tissue slices were embedded in paraffin and processed for histological studies (HE staining). The slices (5 μm) were analyzed under optic microscopy for PMN cell counting (mainly neutrophils), in at least 6 fields, with the Image J software (NIH, USA).

### Immunohistochemistry analyses for TNF-α, iNOS, COX-2, HDAC, and NF-kB

For immunohistochemistry assays, the streptavidin-biotin-peroxidase method was used. Slices from edematous paws from mice subjected to the formalin test were immersed in buffered formalin for 24 h, followed by 70% alcohol solution (24 h), and insertion in paraffin blocks. The sections were then deparaffinized, dehydrated in xylol and ethanol, and immersed in 0.1 M citrate buffer (pH 6) under microwave heating (Panasonic Perfect NN-ST571W, Brazil), for 18 min, for antigen recovery. After cooling at room temperature for 20 min, the sections were washed with phosphate-buffered saline (PBS) solution, followed by a 15-min blockade of endogenous peroxidase with a 3% H_2_O_2_ solution. The sections were incubated overnight (4°C) with rabbit primary antibodies (anti-TNF-α, anti-iNOS, anti-COX-2, anti-NF-kB, and anti-HDAC) diluted in PBS-BSA, according to the manufacturers' instructions. On the next day, the sections were washed in PBS and incubated for 30 min with the secondary biotinylated rabbit antibody (anti-IgG), 1:200 dilution in PBS-BSA. After washing in PBS, the sections were incubated for 30 min at room temperature with the conjugated streptavidin peroxidase complex (ABC Vectastain^®^ complex, Vector Laboratories, USA). After another washing with PBS, the sections were stained with 3,3’ diaminobenzidine-peroxide (DAB) chromophore, counter-stained with Mayer hematoxylin, dehydrated, and mounted on microscope slides for analyses. The data were quantified by the Image J software (NIH, USA). As negative controls, the paw sections from all groups were processed at the same manner, except for the absence of the primary antibodies.

### Statistical analyses

Data are reported as means±SE. The immunohistochemical results (optical microscopy images) were quantified by the Image J software and are reported as absorbance. All the data were statistically analyzed by one-way ANOVA followed by the Tukey multiple comparison test. The significance level was set at P<0.05.

## Results

### FJNB ^1^H NMR spectrum and structural-chemical analyses

**Figure 1:**
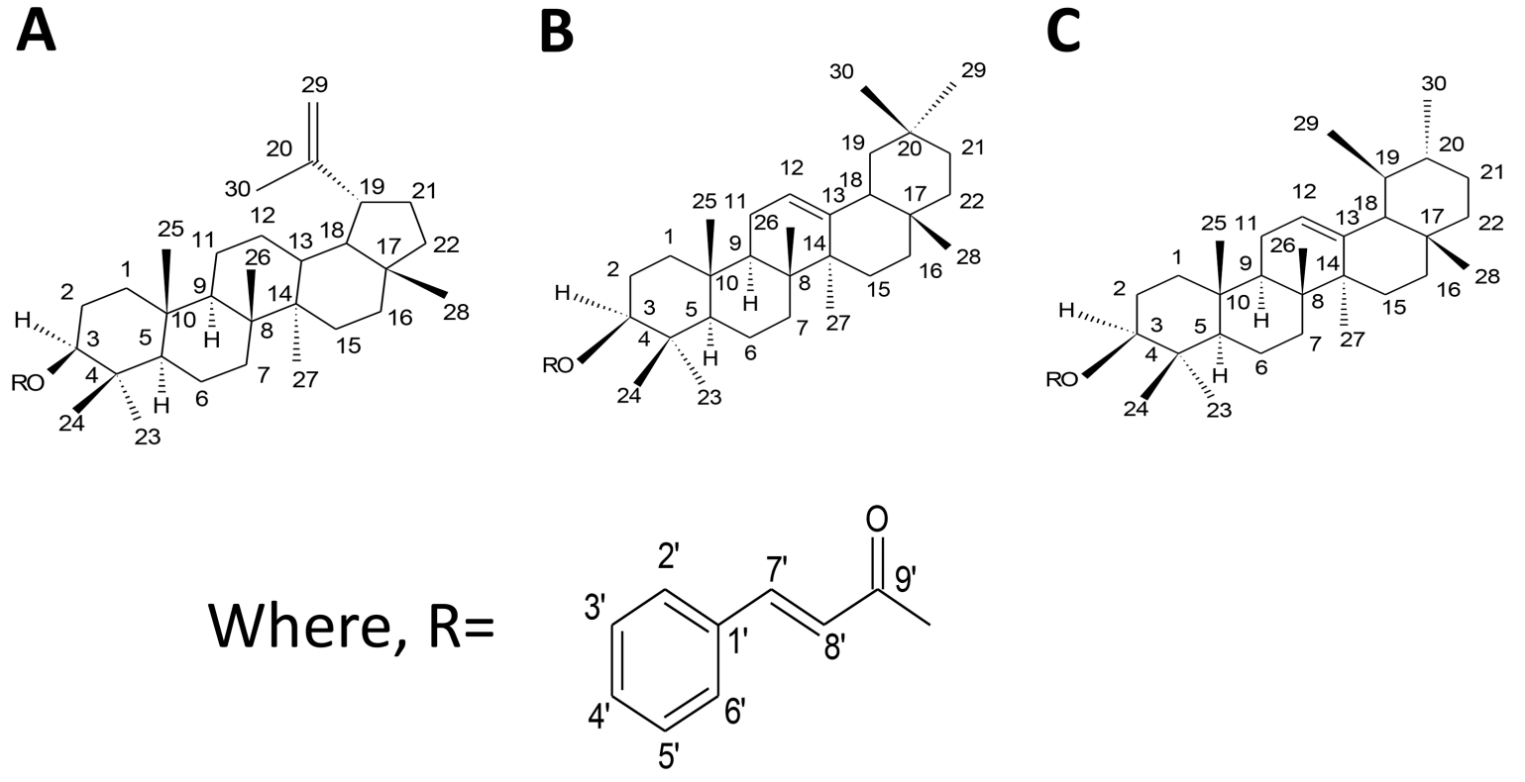
Structural representations for cinnamoyloxy derivatives of lupeol (**A**), β-amyrin (**B**), and α-amyrin (**C**) identified in FJNB from *H. drasticus.*

The ^1^H NMR spectrum of FJBN showed several simple signals in the region of δ 0.8 to 1.0, characteristic of methyl groups on non-hydrogenated carbon of triterpenoids. It also showed signs in δ 7.41 (m) and 7.69 (m) corresponding to benzene hydrogens, supporting the hypothesis that the hydroxyl groups of carbon 3 of the triterpene units were converted into cinnamoyloxy groups. The signals at δ 8.02 (d, 16.0) and δ 6.85 (d, 16.0) shown in the ^1^H NMR spectrum corroborate the suggestion. The ^13^C NMR spectrum of FJBN showed signals in δ 145.11 (C-7’) and δ 167.22 (C-9’) characteristic of olefinic carbons. The suggestion of the lupane skeleton can be attributed to signs δ 81.41; 81.18 (C-3), referring to the oxymethylcarbons of the mixture with hydroxyl substitution. In addition to the signals in δ 110.45 (C-29) and δ 151.48 (C-20), both characteristics of double bond in compounds with lupane skeleton, which along with the ethyl group signal in δ 19.92 (C-30), confirmed the presence of the isopropenyl group (Figure 1).

Also in the ^1^H NMR spectrum, the probable singlet in δ 1.67 (H-30) points to the ethyl group attached to the sp^2^carbon, while the signals in δ 4.69 and 4.70 can be attributed to the olefinicgeminal hydrogens (H-29a and H-29b). This information suggested the presence of a lupeol derivative. Further analysis (extended spectra) of ^1^H and ^13^C NMR of FJBN also allowed to identify the presence of the α- and β-amyrin isomers. Signals in δ 120.07 (C-12) and δ 145.72 (C-13) suggested olefinic carbons of the mixture of the pentacyclic isomers corresponding to the β-amyrin derivative, which has an oleanane type skeleton. On the other hand, the signals in δ 125.22 and δ 40.36 indicated the α-amyrin derivative, which has a basic ursane skeleton. The correlations described, when compared with previous data, allowed the first report of the presence of triterpenic derivatives lupeol, α-amyrin, and β-amyrin coupled to the cinnamoyloxy groups identified in the latex of *H. drasticus.*

### FJNB inhibited the carrageenan-induced paw edema in rats

**Figure 2:**
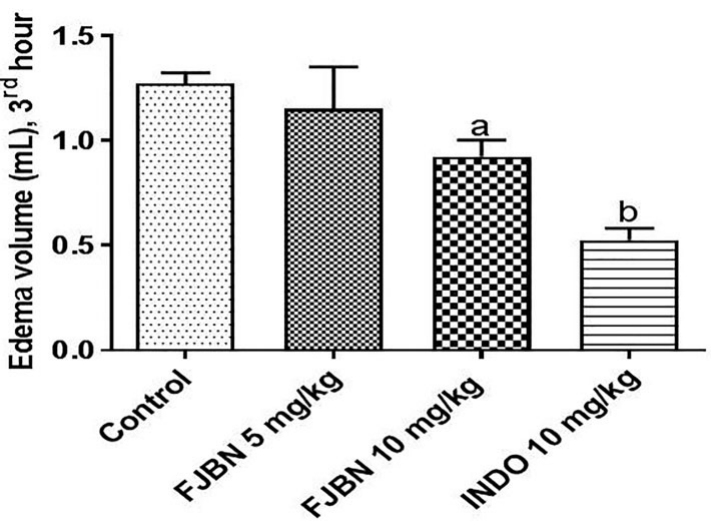
The triterpene-rich fraction from *Himatanthus drasticus* (FJNB) inhibited the carrageenan-induced paw edema in rats at the 3rd h that corresponds to the maximum effect. Indomethacin (INDO) was used as reference. Data are reported as means±SE. ^a^P=0.0402 *vs* Control; ^b^P<0.0001 *vs* Control (one-way ANOVA and Tukey’s test for multiple comparisons).

Inhibition of edema of 10 and 25% were exhibited by FJNB, at the doses of 5 and 10 mg/kg, respectively, compared with the control group at the 3rd h, which corresponds to a maximal effect. Indomethacin (10 mg/kg), used as reference, caused a 59% inhibition (Figure 2).

### FJNB inhibited both the 1st (neurogenic) and the 2nd (inflammatory) phases of the formalin test in mice

**Figure 3:**
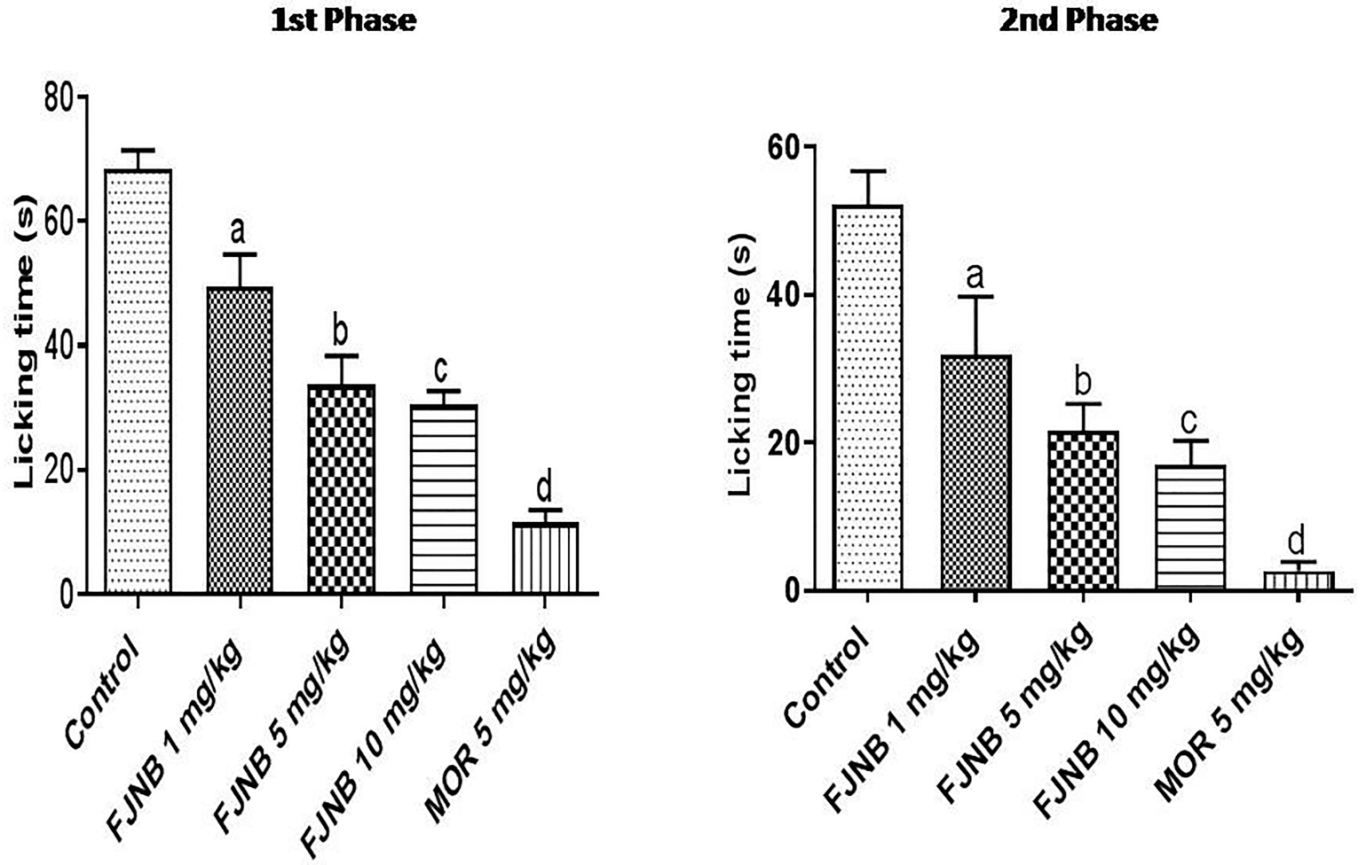
The triterpene-rich fraction from *Himatanthus drasticus* (FJNB) inhibited the 1st (neurogenic) and 2nd (inflammatory) phases of the formalin test in mice. Morphine (MOR) was used as reference. Data are reported as means±SE. 1st Phase: ^a^P<0.05, ^b^P<0.0001, ^c^P<0.0001, ^d^P<0.0001 *vs* Control. 2nd Phase: ^a^P<0.05, ^b^P=0.0014, ^c^P<0.0001, ^d^P<0.0001 *vs* Control (one-way ANOVA and Tukey’s test for multiple comparisons).

*S*ignificant effects were demonstrated with FJNB at the doses of 1, 5, and 10 mg/kg, which caused inhibitions of 27, 49, and 53%, respectively, in the 1st phase, and 37, 50, and 67%, respectively, in the 2nd phase, compared with the control group. Morphine (5 mg/kg), used as reference, caused 84 and 92% inhibitions in the 1st and 2nd phases of the test, respectively (Figure 3).

### FJNB inhibited the PMN migration in the edematous paw

**Figure 4:**
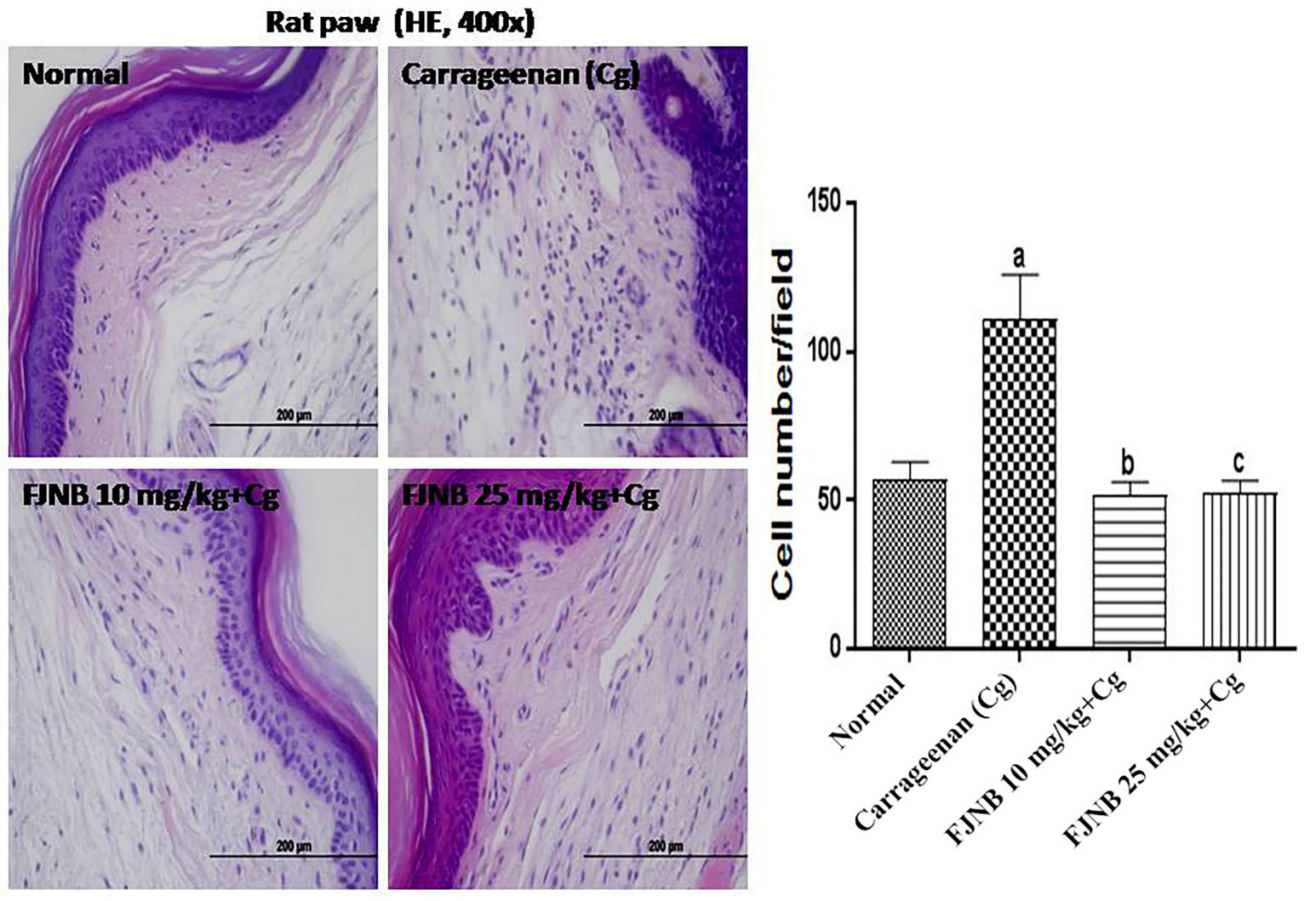
Photomicrographs of hematoxylin/eosin staining from paws of rats subjected to the carrageenan-induced edema test (×400 magnification, scale bar=200 µm). The triterpene-rich fraction from *Himatanthus drasticus* (FJNB) inhibited polymorphonuclear cell migration from inflamed paws of rats. Data are reported as means±SE. ^a^P<0.001 *vs* Normal; ^b^P<0.001 and ^c^P<0.001 *vs* carrageenan (one-way ANOVA and Tukey’s test for multiple comparisons).

The number of PMN cells (mainly neutrophils) was increased by almost 2 times in the edematous paw in the control group, compared with the normal rats. The number of PMN cells in the edematous paws went to values near or even lower than those from normal rats after FJNB treatments with the doses of 10 and 25 mg/kg (Figure 4).

### FJNB decreased the immunoreactivities for TNF-alpha, iNOS, COX-2, HDAC, and NF-kB in the inflamed paw

**Figure 5:**
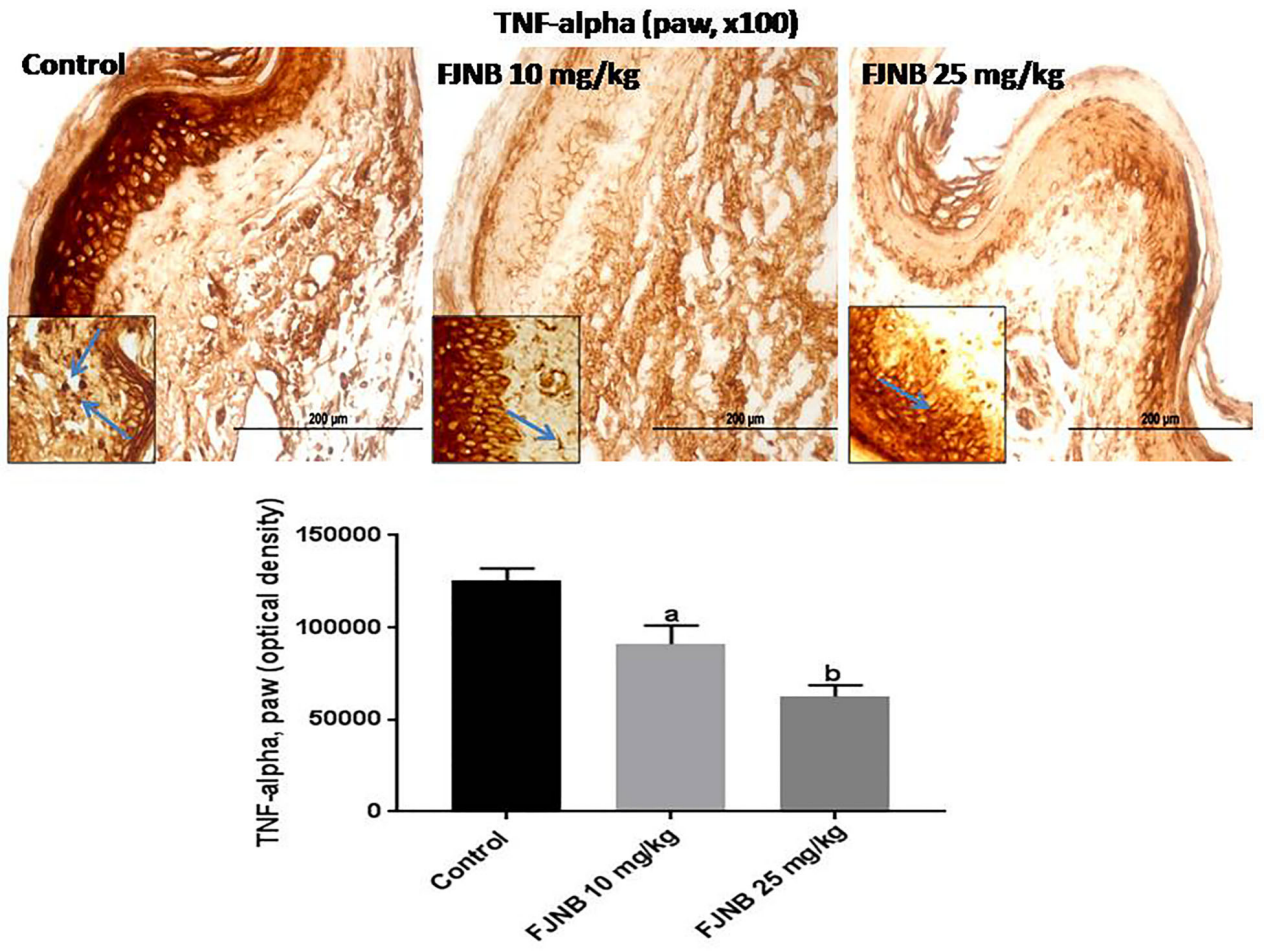
The triterpene-rich fraction from *Himatanthus drasticus* (FJNB) inhibited the immunostaining for tumor necrosis factor (TNF)-alpha in the inflamed paws (Controls) from the formalin test, in mice. Representative photomicrographs (×100 magnification, scale bar, 200 µm). The inserts were taken at ×400 magnification and blue arrows show polymorphonuclear cells. Data are reported as means±SE for 3 different animals. ^a^P<0.05 and ^b^P<0.001 *vs* Control (one-way ANOVA and Tukey’s test for multiple comparisons).

**Figure 6:**
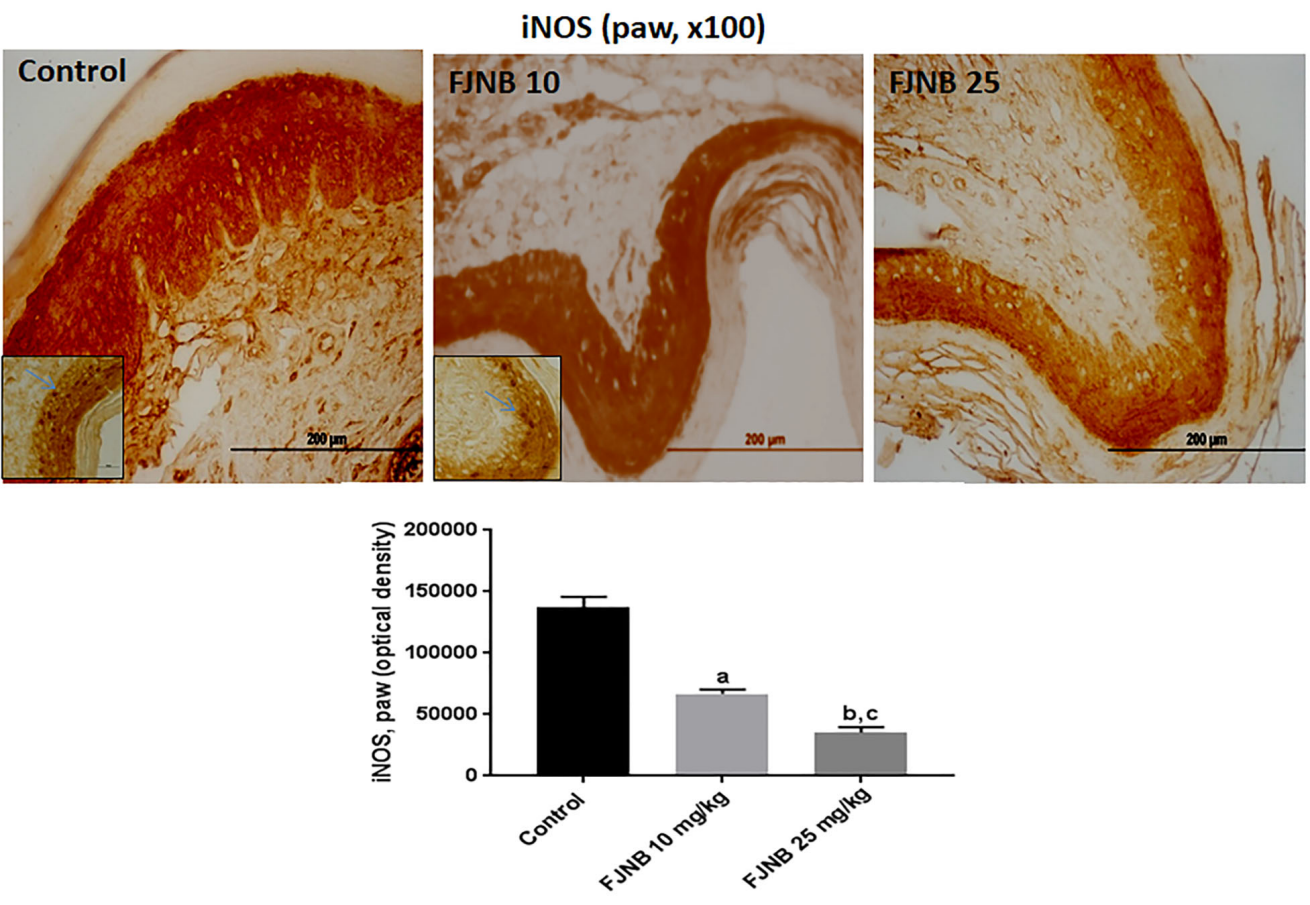
The triterpene-rich fraction from *Himatanthus drasticus* (FJNB) inhibited the immunostaining for inducible nitric oxide synthase (iNOS) in inflamed paws (Controls) from the formalin test in mice. Representative photomicrographs (×100 magnification, scale bar, 200 µm). The inserts were taken at ×400 magnification and blue arrows show polymorphonuclear cells. Data are reported as means±SE for 3 different animals. ^a^P<0.05 and ^b^P<0.001 *vs* Control; ^c^P<0.05 *vs* FJNB 10, (one-way ANOVA and Tukey test for multiple comparisons).

**Figure 7:**
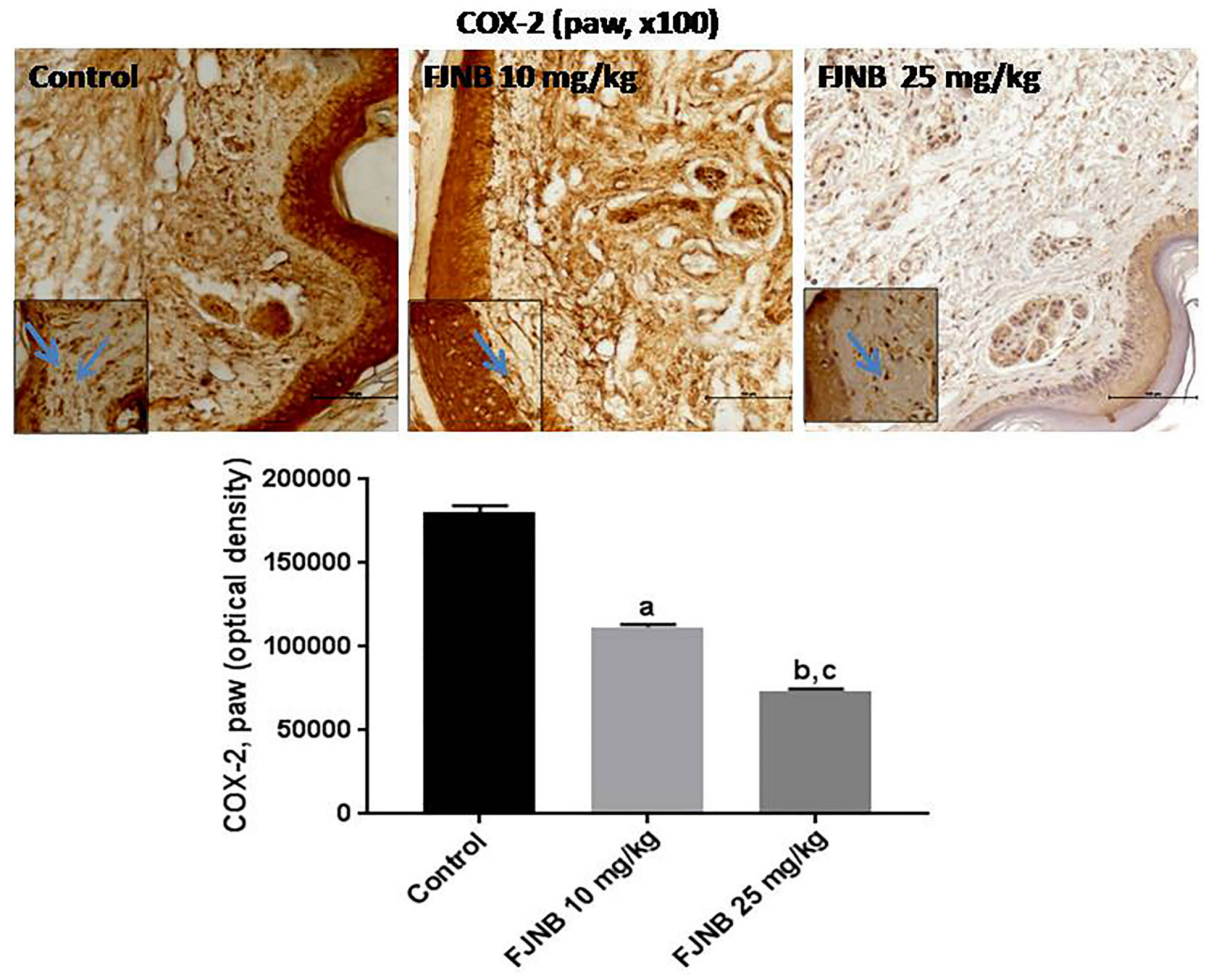
. The triterpene-rich fraction from *Himatanthus drasticus* (FJNB) inhibits the immunostaining for COX-2 in the inflamed paws (Controls) from the formalin test, in mice. Representative photomicrographs (×100 magnification, scale bar, 200 µm). The inserts were taken at ×400 magnification and blue arrows show polymorphonuclear cells. Data are reported as means±SE for 3 different animals. ^a,b^P<0.0001 *vs* Control; ^c^P<0.0001 *vs* FJNB 10 (one-way ANOVA and Tukey’s test for multiple comparisons).

**Figure 8:**
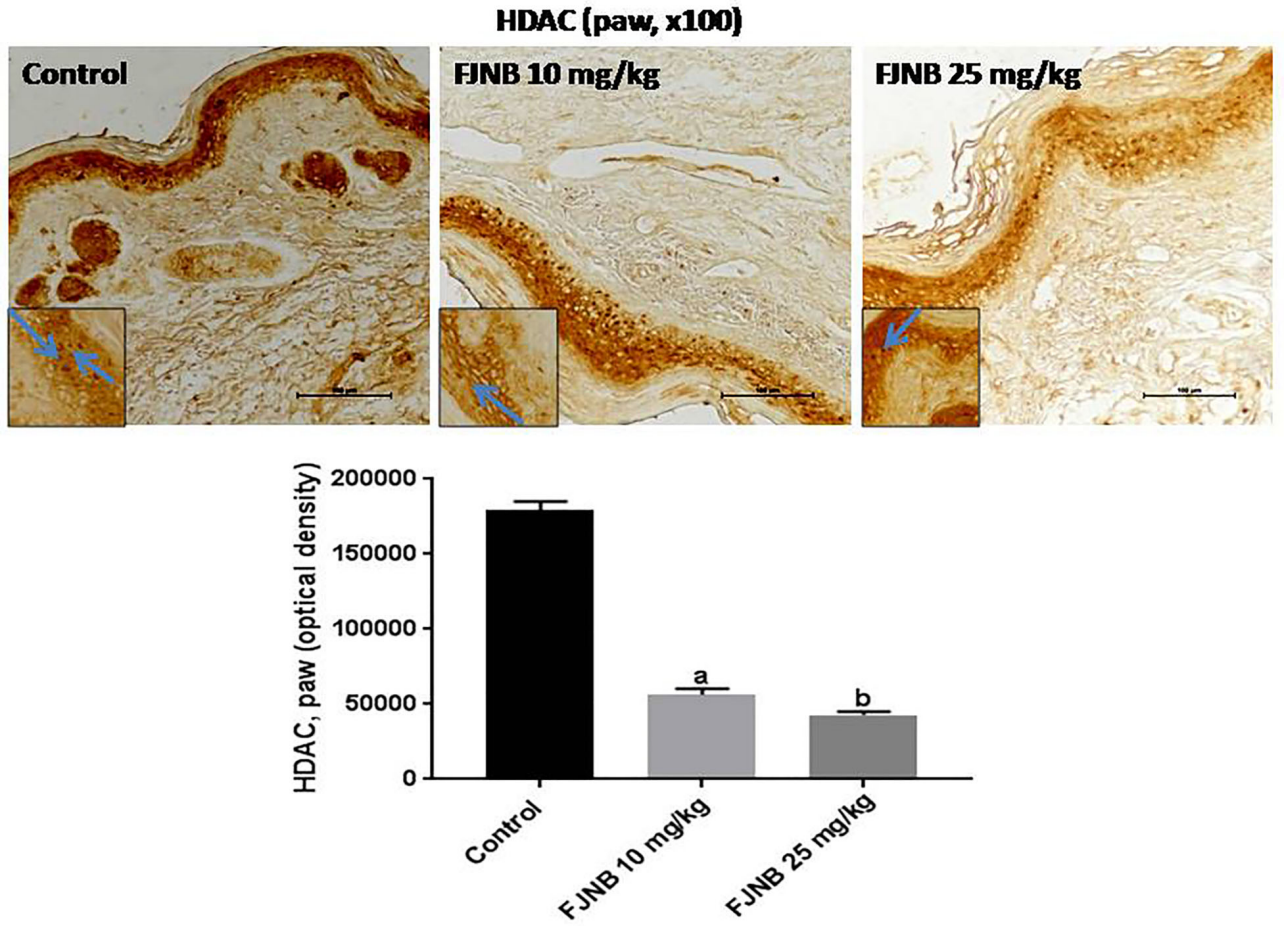
The triterpene-rich fraction from *Himatanthus drasticus* (FJNB) inhibited immunostaining for histone deacetylase (HDAC) in inflamed paws (Controls) from the formalin test in mice. Representative photomicrographs (×100 magnification, scale bar, 200 µm). The inserts were taken at ×400 magnification and blue arrows show polymorphonuclear cells. Data are reported as means±SE for 3 different animals. ^a,b^P<0.0001 *vs* Control (one-way ANOVA and Tukey’s test for multiple comparisons).

**Figure 9:**
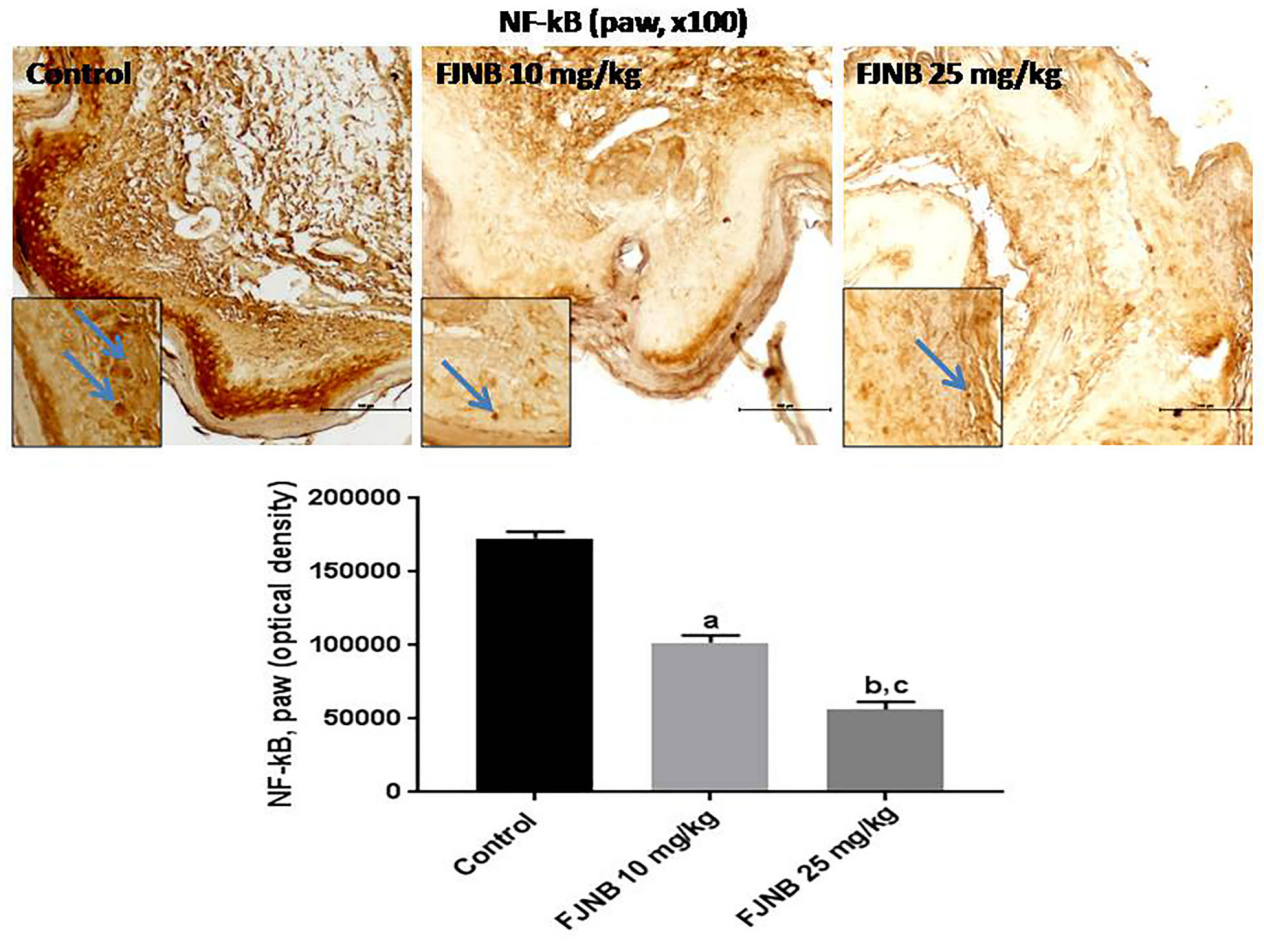
The triterpene-rich fraction from *Himatanthus drasticus* (FJNB) inhibited the immunostaining for NF-kB in the inflamed paws from the formalin test in mice. Representative photomicrographs (×100 magnification, scale bar, 200 µm). The inserts were taken at ×400 magnification and blue arrows show polymorphonuclear cells. Data are reported as means±SE for 3 different animals. ^a,b^P<0.0001 *vs* Control; ^c^P<0.001 *vs* FJNB 10 (one-way ANOVA and Tukey’s test for multiple comparisons).

**Figure 10:**
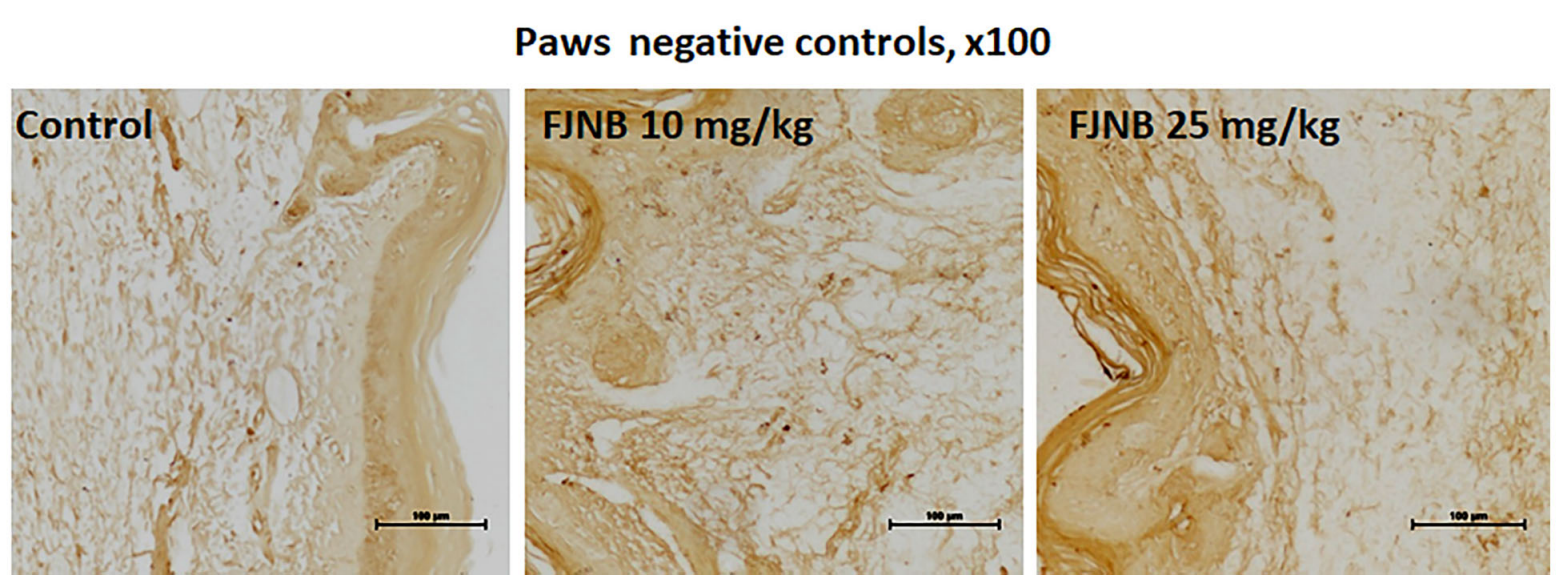
Representative photomicrographs (×100 magnification, scale bar, 100 µm) of negative controls (inflamed paws, after the formalin test) of the three tested groups. The immunohistochemical assays were performed in the absence of the primary antibodies.

We showed that FJNB significantly reduced TNF-alpha immunostaining, compared with the control group. Thus, reductions of 28 and 50% were observed with FJNB at the doses of 10 and 25 mg/kg, respectively (Figure 5). A similar pattern, although with higher inhibitions, was demonstrated in immunohistochemistry for iNOS, with reductions of 52 and 74% after treatments with FJNB at the same doses, compared with the control group (Figure 6). The groups subjected to immunohistochemistry for COX-2 showed immunoreactivity reductions of 42 and 74%, after the same treatment, in relation to the control group (Figure 7). Significant reductions in HDAC immunostainings were observed in the group treated with FJNB 10 (69%) and FJNB 25 groups (76%), compared with the control group (Figure 8). A decrease of 41% in the NF-kB immunostaining was shown by the group treated with 10 mg/kg FJNB relative to the control group. In addition, a significant decrease (67%) in the NF-kB immunoreactivity was observed after the 25 mg/kg FJNB treatment compared with controls (Figure 9). The images from negative controls with assays performed in the absence of primary antibodies are shown in Figure 10.

## Discussion

In the present study, we investigated the antinociceptive and anti-inflammatory actions of the triterpene-rich fraction (FJNB) from the *H. drasticus* latex. We showed that FJNB (10 mg/kg) presented potent antinociceptive and anti-inflammatory activities, as demonstrated in the 2nd phase of the formalin test in mice and in the carrageenan-induced paw edema test in rats. In addition, the edematous paws from animals subjected to the carrageenan-induced edema test were processed for histological studies and showed a reduction in PMN cells migration in the FJNB-treated group, compared with the control group.

The formalin test in mice is a valid and reliable model of nociception, sensitive to various classes of analgesic drugs and involving moderate and continuous pain generated by the injured tissue. The early phase (neurogenic) is caused mainly by C-fiber activation, while the late phase (inflammatory) is dependent upon the combination of an inflammatory reaction in the peripheral tissue and functional changes in the dorsal horn of the spinal cord (11). Furthermore, there are several mediators involved in inflammation, such as histamine, serotonin, bradykinin, and prostaglandins (PG), which are involved in increased vascular permeability. Another mediator in acute inflammation is nitric oxide (NO). Carrageenan-induced paw edema displays all these biochemical and cellular features (12).

Inflammation is a physiological body response against any harmful stimulus, leading to the activation of inflammatory cells such as neutrophils, eosinophils, mononuclear phagocytes, and macrophages. Furthermore, these inflammatory cells, especially macrophages, secrete increased amounts of NO, iNOS, PGE2, COX-2, and cytokines, such as TNF-alpha (13,14).

Previously (6), we showed that the pentacyclic triterpene lupeol acetate isolated from *H. drasticus* latex presents an anti-inflammatory action, as demonstrated in experimental models of acute inflammation. Pentacyclic triterpenes are secondary metabolites largely present in plants (fruit peel, leaves, and stem bark) and are promising compounds for the development of multi-target bioactive drugs (15). The anti-inflammatory effect of lupeol probably involves the opioid system, as indicated by the blockade of its effect by the opioid antagonist naloxone. Lupeol effects were potentiated by pentoxifylline, a known TNF-alpha inhibitor. In addition, lupeol also decreased iNOS immunostaining.

In the present work, we showed that FJNB reduced immunopositive cells for iNOS. Furthermore, COX-2 and TNF-α immunostainings were also reduced, effects already observed with triterpenes from other species, including the triterpene beta-amyrin, known to be present in our FJNB fraction (16–18). TNF-alpha has been shown to play important roles in both inflammatory and neuropathic hyperalgesia, and the intraplantar injection of complete Freund's adjuvant in adult rats resulted in a significant elevation of TNF-alpha, IL-1β, and nerve growth factor levels in the inflamed paw (19). Thus, inhibition of TNF-alpha could certainly contribute to the anti-inflammatory effect of FJNB, as observed by us in the present work.

Pentacyclic triterpenes are well recognized as analgesic anti-inflammatory drugs (20–25). Recently (26), lupeol was shown to inhibit the LPS-induced activation of glial cells and to decrease the LPS-induced generation of TNF-alpha, iNOS, and IL-1β, evidencing its therapeutic potential for inflammatory disorders. Beta-amyrin, another pentacyclic triterpene, was shown to inhibit LPS-induced PGE2, IL-6 secretion, and NF-kB action on human mononuclear cells *in vitro* (27). In addition, beta-amyrin exhibited anti-fibrotic, anti-inflammatory, and anti-apoptotic effects on a model of hepatic fibrosis in rats (28).

Pentacyclic triterpenes are a group of promising secondary plant metabolites, and the potential of those belonging to the lupane, oleanane, or ursane groups for treating cancer are especially important (29). They act through a variety of mechanisms and the majority of them inhibit the NF-κB signaling pathway. Lupeol is a multi-target agent with immense anti-inflammatory potential, targeting key molecular pathways that involve NF-κB, among others, in a variety of cells (30). Recently (31), lupeol was shown to block the NF-κB signaling in human epithelial cells and murine macrophages, and to attenuate experimental murine colitis.

In the present study, we showed that FJNB, besides inhibiting iNOS, COX-2, and TNF-alpha, also inhibited NF-kB. This nuclear factor is a pro-inflammatory signaling pathway with an important role in the expression of pro-inflammatory cytokines, including TNF-alpha (32). Furthermore, potent anti-inflammatory activity in the isomeric mixture α- and β-amyrin has been demonstrated by us (33) and others (34). Thus, we showed that the anti-inflammatory activity of these triterpenes was potentiated by both indomethacin and thalidomide, suggesting a potential involvement of prostaglandins and TNF-alpha inhibitions (35). In addition, this isomeric mixture suppressed inflammatory cytokines and COX-2 levels, possibly via inhibition of NF-kB and CREB-signaling pathways, in a model of colitis (36).

However, important findings of the present study were the inhibitions of HDAC and NF-kB immunostainings by FJNB, demonstrated here for the first time. *In vitro* and *in vivo* data indicate that HDAC inhibitors may be anti-inflammatory due to their effects on cell death, acting through acetylation of non-histone proteins (37). Histone acetylation has been demonstrated to have a fundamental role in numerous inflammatory diseases (38). Thus, pharmacological modulators of HDAC activity possess potent anti-inflammatory effects in experimental animal models as well as in the clinic, and some HDAC inhibitors have already shown therapeutic action in animal models of several inflammatory diseases.

Furthermore, the activation of the NF-kB transcription factor by nuclear translocation of cytoplasmic complexes plays an important role in inflammation by inducing the transcription of pro-inflammatory genes, including those encoding cytokines (39). Besides serving as a pivotal mediator of inflammatory responses, it regulates multiple aspects of innate and adaptive immune functions, and the dysregulation of NF-kB activation contributes to the pathogenic process of various inflammatory diseases (40). In addition, the resolution of inflammation is an active process that involves the suppression of pro-inflammatory gene expression and of leukocyte migration and activation, followed by cells apoptosis and phagocytosis that are determinants for a successful outcome.

FJNB showed α- and β-amyrin and lupeol derivatives, such as pentacyclic triterpenes in its chemical composition, probably responsible, at least in part, for its observed effects. FJNB, by inhibiting oxidative stress, inflammatory enzymes, pro-inflammatory cytokines, as TNF-alpha and mainly HDAC and NF-kB, is a promising candidate for submission to translation studies, focusing on therapeutic alternatives for the treatment of inflammation-associated diseases.
